# Carboxypeptidase B blocks ex vivo activation of the anaphylatoxin-neutrophil extracellular trap axis in neutrophils from COVID-19 patients

**DOI:** 10.1186/s13054-021-03482-z

**Published:** 2021-02-08

**Authors:** Yue Zhang, Kai Han, Chunjing Du, Rui Li, Jingyuan Liu, Hui Zeng, Liuluan Zhu, Ang Li

**Affiliations:** 1 Beijing Key Laboratory of Emerging Infectious Diseases, Institute of Infectious Diseases, Beijing Ditan Hospital, Capital Medical University, Beijing, 100015 China; 2 Department of Critical Care Medicine, Beijing Ditan Hospital, Capital Medical University, Beijing, 100015 China

**Keywords:** COVID-19, Neutrophil extracellular trap, anaphylatoxin, Carboxypeptidase B

## Abstract

**Background:**

Thrombosis and coagulopathy are highly prevalent in critically ill patients with COVID-19 and increase the risk of death. Immunothrombosis has recently been demonstrated to contribute to the thrombotic events in COVID-19 patients with coagulopathy. As the primary components of immunothrombosis, neutrophil extracellular traps (NETs) could be induced by complement cascade components and other proinflammatory mediators. We aimed to explore the clinical roles of NETs and the regulation of complement on the NET formation in COVID-19.

**Methods:**

We recruited 135 COVID-19 patients and measured plasma levels of C5, C3, cell-free DNA and myeloperoxidase (MPO)-DNA. Besides, the formation of NETs was detected by immunofluorescent staining and the cytotoxicity to vascular endothelial HUVEC cells was evaluated by CCK-8 assay.

**Results:**

We found that the plasma levels of complements C3 and MPO-DNA were positively related to coagulation indicator fibrin(-ogen) degradation products (C3: *r* = 0.300, *p* = 0.005; MPO-DNA: *r* = 0.316, *p* = 0.002) in COVID-19 patients. Besides, C3 was positively related to direct bilirubin (*r* = 0.303, *p* = 0.004) and total bilirubin (*r* = 0.304, *p* = 0.005), MPO-DNA was positively related to lactate dehydrogenase (*r* = 0.306, *p* = 0.003) and creatine kinase (*r* = 0.308, *p* = 0.004). By using anti-C3a and anti-C5a antibodies, we revealed that the complement component anaphylatoxins in the plasma of COVID-19 patients strongly induced NET formation. The pathological effect of the anaphylatoxin-NET axis on the damage of vascular endothelial cells could be relieved by recombinant carboxypeptidase B (CPB), a stable homolog of enzyme CPB2 which can degrade anaphylatoxins to inactive products.

**Conclusions:**

Over-activation in anaphylatoxin-NET axis plays a pathological role in COVID-19. Early intervention in anaphylatoxins might help prevent thrombosis and disease progression in COVID-19 patients.

## Background

As declared by the World Health Organization, the global outbreak of coronavirus disease 2019 (COVID-19) has posed an unprecedented health crisis and caused more than 1.5 million deaths(https://covid19.who.int/). As the pandemic continues, mounting evidence implicates thrombosis and coagulopathy in a fatal outcome in COVID-19 patients [1, 2]. The latest data reported that thrombotic complications occur in up to 49% of patients with COVID-19 admitted to the intensive care unit (ICU) [3]. Autopsies further provide direct evidence of pulmonary embolism in patients with COVID-19 [4, 5]. Of note, immunothrombosis, the direct interaction of activated leukocytes with platelets and plasma coagulation factors in the innate immune response [6], has been demonstrated to contribute to the thrombotic events of coagulopathy [7]. Besides, the formation of neutrophil extracellular traps (NETs), which are composed of extracellular DNA decorated with granule proteins released by activated neutrophils, was identified as a leading cause and core component of immunothrombosis [8–10]. Hence, it is crucial to reveal the mechanisms causing NET formation in exploring more efficient therapeutic approaches to combat COVID-19.

Complement is a major component of the innate immune system involved in defending against foreign pathogens through complement fragments [11]. During the complement activation, the central component C3 is cleaved into C3a and C3b. C3b binds to C3 convertase to form C5 convertase, which proteolytically cleaves C5 into C5a and C5b [12, 13]. The anaphylatoxin C3a and C5a function as potent activators for neutrophil migration, cytokine production, platelet-leukocyte aggregation, and NET release, by binding to receptors C3aR and C5aR on the surface of neutrophils [12, 14–16]. Multiple studies have illustrated that complement-induced over-activation of neutrophils is involved in the pathogenesis of acute respiratory distress syndrome (ARDS) and fatal viral infections [17–19]. More importantly, complement C5a- and C3a-mediated NET formation has recently been demonstrated to be the key driver in COVID-19 immunothrombosis [20]. Thus, complements are important soluble mediators that bridge inflammation and thrombosis in COVID-19 and other infectious diseases.

Of note, C3a and C5a have carboxyl-terminus containing arginine residues that are critical for optimal activity [21]. Carboxypeptidases are capable of controlling the activity of anaphylatoxins by cleaving off a C-terminal arginine residue to yield arginine derivatives (C3a_des-Arg_ and C5a_des-Arg_) [12]. The resulting C5a_des-Arg_ retains 1–10% of the inflammatory activity of C5a, and C3a_des-Arg_ is devoid of any pro-inflammatory activity [22]. Recently, Carboxypeptidase B2 (CPB2, encoded by human *CPB2* gene) was demonstrated to be an important regulator in reducing inflammatory response and organ damage by degrading plasma anaphylatoxins [23–25].

In this study, we found that the plasma levels of complements and NETs were associated with disease severity in COVID-19. More importantly, we demonstrated that recombinant CPB could reduce the NET formation by degrading anaphylatoxin C3a and C5a. These findings may shed new light on a potential therapeutic strategy for COVID-19 by targeting the anaphylatoxin-NET axis.

## Methods

### Patient and sample collection

The prospective study included 135 patients with confirmed diagnosis of COVID-19 who admitted to Beijing Ditan Hospital from January 20^th^, 2020 to April 27^th^, 2020. The following patients were excluded from the present study: 1. Age < 18; 2. Gestation; 3. Patients with any immunodeficiency such as neutrophilia, neutropenia, malignant tumor, using of immunosuppressants for more than 1 week; 4. The time from onset to admission is more than 2 weeks; 5. Dropout patients; 6. Patients or their guardians do not want to be included in the study. According to the inclusion/exclusion criteria, 148 patients were enrolled in the study cohort, and 13 of them quitted before the study was completed. According to the guidelines on the diagnosis and treatment protocol for novel coronavirus pneumonia (trial version 7) [26] released by National Health Commission & National Administration of Traditional Chinese Medicine of China, the classification of COVID-19 are as follows: Mild: Clinical symptoms from mild fever, respiratory tract to pneumonia manifestation. Severe: Meeting any one of the following should be treated as severe cases, including respiratory distress, respiratory rate ≥ 30 breaths/min; oxygen saturation ≤ 93% at rest; and PaO_2_/FiO_2_ ≤ 300. In severe group, 31 patients were admitted to ICU, 15 cases received mechanical ventilation and 1 of them deceased. In severe group, 11 of 41 patients (26.8%) were first diagnosed as mild/moderate and then crossed over to severe COVID-19. In treating with coagulation disorders, 22 severe patients received enoxaparin sodium, 1 severe patient received low-molecular-weight heparin sodium and 1 severe patient received dabigatran treatment. The other 17 severe patients and all the mild patients did not receive anticoagulant therapy. Twenty-five healthy donors matched to the age and sex of mild COVID-19 patients were enrolled. Three volunteers donated their peripheral neutrophils for in vitro experiments.

The first sample of each patient was collected within 24 h after admission. Then, the blood was taken once a week until discharge from hospital. Blood samples were collected by venipuncture into ethylenediaminetetraacetic acid tubes. Plasma was separated from blood by centrifugation at 450 × g (break off) for 10 min at room temperature. Plasma samples were divided into small aliquots and stored at − 80 °C until the time of testing. The study was approved by Committee of Ethics at Beijing Ditan Hospital, Capital Medical University, Beijing, China. The approval number is JDLKZ(2020)D(036)-01.

### Quantification of MPO-DNA and cfDNA

Cell-free DNA in plasma was quantified using the Quant-iT PicoGreen dsDNA Assay Kit (Invitrogen, Carlsbad, CA, USA) according to the manufacturer’s instruction. MPO-DNA complexes were quantified similarly to what has been previously described [27]. In brief, a capture antibody against MPO was coated on a 96-well flat-bottom plate at 1:200 (Abcam, Cambridge, MA, USA), and the amount of MPO-bound DNA was quantified using the Quant-iT PicoGreen dsDNA assay as described above.

### Quantification of C5 and C3

Plasma levels of C5 (including the sum of C5 and C5a) and C3 (including the sum of C3, C3a and C3b) were detected using Human Complement C5 ELISA Kit and Human Complement C3 ELISA Kit (Abcam) according to the manufacturer’s instruction.

### Immunofluorescence Staining

NETs were detected by immunofluorescence staining as previously reported [28]. Neutrophils were fixed, permeabilized and blocked after 3-h in vitro culture or stimulation. Cells were incubated with antibody against histone H3 citrulline R2 + R8 + R17 (H3Cit; Abcam) at 1:400 for 1 h at 37℃, followed by secondary antibody coupled with Alexa Fluor Dyes (Invitrogen) at 1:1000 for 1 h at room temperature. DNA was stained using 4′,6-diamidino-2-phenylindole (DAPI; Cell Signaling Technology, London, UK) at 1:2000 for 8 min at room temperature. Images were obtained with a confocal fluorescence microscope (Zeiss LSM 510 META; Carl Zeiss, Thornwood, NJ, USA). NETs were identified as structures positive for both histone H3Cit and DAPI staining.

### Neutrophil isolation, in vitro culture and stimulation

Blood samples from healthy donors were collected into ethylenediaminetetraacetic acid tubes as described above for plasma separation. The anticoagulated blood was then fractionated by density-gradient centrifugation using Percoll (Stemcell Technologies, Vancouver, Canada). Neutrophils were further purified by dextran sedimentation of the red blood cell layer before lysing residual red blood cells with sodium chloride. Neutrophil preparations were at least 95% pure as confirmed by nuclear morphology.

Purified neutrophils were resuspended in RPMI-1640 medium supplemented with heat-inactivated 5% fetal bovine serum and 2 mM L-glutamine. Neutrophils were seeded into 96-well plate (5 × 10^4^/well) for supernatant detection and 24-well plate (2 × 10^5^/well) with polylysine-coated coverslips for NET immunofluorescence staining. Cells were rested for 1 h at 37 °C and 5% CO_2_ followed by replacement with plasma from HCs or COVID-19 patients at a final concentration of 5% in RPMI-1640 medium for 3 h according to previous report [20]. Recombinant CPB (YaxinBio, Shanghai, China) was used at 100 μg/ml to digest C3a and C5a in the plasma at 37 °C for 30 min prior to neutrophil stimulation. One hour prior to neutrophil stimulation, 100 μg/ml anti-human C3a (Merck, Darmstadt, Germany) or 10 μg/ml anti-human C5a antibody (R&D Systems, Minneapolis, MN, USA) were added into the culture system for neutralizing C3a or C5a in the plasma. Three hours later, the supernatant was collected to quantify MPO-DNA content. The results were calculated by deducting the background levels of MPO-DNA in the plasma.

### Preparation of NET-conditioned medium and cell viability assay

Neutrophils from healthy donors were cultured with RPMI-1640 medium supplied with 5% plasma from patients with COVID-19 as described above. The peptidylarginine deiminase inhibitor Cl-amidine was added at 200 μM for blocking NET formation. Three hours later, the supernatant was collected carefully and used as NET-conditioned medium. HUVEC cells (3 × 10^3^/well) were seeded into 96-well plate and cultured for 24 h. Cells culture media were replaced with 100 μl NET-conditioned media or new cell culture media as control for 24 h. Cell viability assay was performed using a cell counting kit 8 (CCK‐8) (Dojindo, Kumamoto, Japan) as per the manufacturer's protocol. Absorbance was detected at 590 nm using a microplate reader. The experiments were performed in sextuplicate.

### Statistical Analysis

All statistical analyses were performed with the SPSS 25.0 statistical package (IBM, Armonk, NY, USA). Values are presented as the mean ± standard deviation for data that were normally distributed or median and interquartile range for data that were not normally distributed for continuous variables and number (%) for categorical variables. The Kolmogorov–Smirnov test was used to inspect the normality and homogeneity of variance of all the data. For two-group comparison, *P* values were derived from the one-way Student *t* test to determine differences between groups with normally distributed data and Mann–Whitney nonparametric test with other data. For multi-group comparison, *P* values were derived from one-way ANOVA (continuous variables) or Chi-square test (categorical variables). For all comparisons, *P* < 0.05 was considered statistically significant.

## Results

### COVID-19 cohort

We recruited 135 confirmed COVID-19 patients admitted to Beijing Ditan Hospital. Among them, 94 (69.6%) were mild cases and 41 (30.4%) were severe cases according to the guidelines on the diagnosis and treatment protocol for novel coronavirus pneumonia (trial version 7) [26] released by National Health Commission & National Administration of Traditional Chinese Medicine of China. Twenty-five age and gender-matched healthy donors (HDs) were enrolled as controls. Demographics and relevant clinical characteristics are reported in Table 1. The study was approved by the Committee of Ethics at Beijing Ditan Hospital, Capital Medical University, Beijing, China.

**Table 1:** Demographics and clinical characteristics of healthy donors and 2 groups of COVID-19 patients

Characteristic	HD	Severity		*P* value (HD vs. Mild)	*P* value (Mild vs. Severe)
		Mild	Severe		
Patient amount	25	94	41	–	–
Age (years)	42 (32, 69)	34.9 ± 15.0	61.8 ± 16.7	**0.001**	** < 0.001**
Male sex, *n* (%)	15 (60)	43 (45.7)	26 (63.4)	0.607	0.059
Complications, *n* (%)					
Hypertension	0	6 (6.4)	16 (39.0)	–	** < 0.001**
Cardiovascular disease	0	0	5 (12.2)	–	**0.001**
Chronic pulmonary disease	0	2 (2.1)	9 (22.0)	–	** < 0.001**
Diabetes	0	1 (1.1)	10 (24.4)	–	** < 0.001**
Hyperlipemia	0	1 (1.1)	2 (4.9)	–	0.167
Chronic kidney disease	0	1 (1.1)	4 (9.8)	–	**0.014**
Immune disorders	0	0	3 (7.3)	–	**0.008**
Others	0	1 (1.1)	1 (2.4)	–	0.543
Hospitalization Period (days)	0	25.5 (17.3, 35.7)	36 (22, 43)	–	**0.004**
SOFA score	–	0 (0, 0)	2 (0, 3.5)	–	** < 0.001**
APACHE II score	–	–	14 (9, 15)	–	–
Laboratory data at admission					
WBC (× 10^9^/L)	6.1 ± 1.1	4.8 (4.1, 6.5)	6.7 (4.8, 8.9)	**0.007**	** < 0.001**
Lymphocyte (× 10^9^/L)	2.2 (2.0, 2.6)	1.6 (1.2, 2.0)	1.0 (0.8, 1.4)	** < 0.001**	** < 0.001**
Neutrophil (× 10^9^/L)	3.4 ± 0.8	2.7 (2.1, 3.8)	5.2 (3.3, 7.9)	**0.045**	** < 0.001**
Monocyte (× 10^9^/L)	0.4 ± 0.1	0.3 (0.2, 0.4)	0.3 (0.2, 0.4)	**0.006**	0.429
Hemoglobin (g/L)	140.6 ± 13.4	137.5 ± 14.4	121.4 ± 18.9	0.431	** < 0.001**
Platelets (× 10^9^/L)	248.1 ± 41	230.5 (182, 278.3)	214 (153, 296)	0.180	0.277
D-dimer (mg/L)	–	0.3(0.1, 0.4)	1.1 (0.6, 2.2)	–	** < 0.001**
CRP (mg/L)	–	1.6 (0.6, 9.4)	47.1 (24.7, 105.3)	–	** < 0.001**
LDH (U/L)	–	195.5 (172.8, 227.6)	341.1 (296.3, 474.5)	–	** < 0.001**
CK (U/L)	–	69.1 (46.4, 105.4)	110.1 (48.1, 175.4)	–	**0.049**
ALT (U/L)	–	19.5 (14, 30.2)	30.4 (17.8, 47.5)	–	**0.008**
AST (U/L)	–	20.2 (15.7, 27)	33.7 (22.1, 51.5)	–	** < 0.001**
TBIL (umol/L)	–	9.6 (7.4, 14.3)	11.7 (8, 17.5)	–	0.171
DBIL (umol/L)	–	3.4 (2.4, 4.7)	4.6 (3.5, 8.7)	–	**0.001**
BUN (mmol/L)	–	4.3 ± 1.1	4.4 (3.7, 6.3)	–	0.439
sCr (umol/L)	–	66.1 (53.4, 78.2)	69.0 (52.8, 84.5)	–	0.470
GFR (ml/min/1.73m^2^)	–	110.9 (99.1, 126.9)	94.2 ± 21.8	–	** < 0.001**

Data were presented as mean ± standard deviation or as median (interquartile range) for continuous variables and number (%) for categorical variables. The different characteristics between two groups were tested by one-way *t* tests (normally distributed continuous variables), Mann Whitney nonparametric test (nonnormally distributed variables) or Chi-square test (categorical variables). A two-sided α of less than 0.05 was considered statistically significant

Abbreviations: SOFA score, sequential organ failure assessment score; APACHE II score, acute physiology and chronic health evaluation II score; WBC, white blood cells; CRP, C-reactive protein; LDH, lactate dehydrogenase; CK, creatine kinase; ALT, alanine aminotransferase; AST, aspartate aminotransferase; TBIL, total bilirubin; DBIL, direct bilirubin; BUN, blood urea nitrogen; sCr, serum creatinine; GFR, glomerular filtration rate

*P* < 0.05 was considered statistically significant and is shown in bold

### The elevated levels of NETs in COVID-19 patients were closely related to respiratory failure and multiple organ dysfunction

**Fig. 1:**
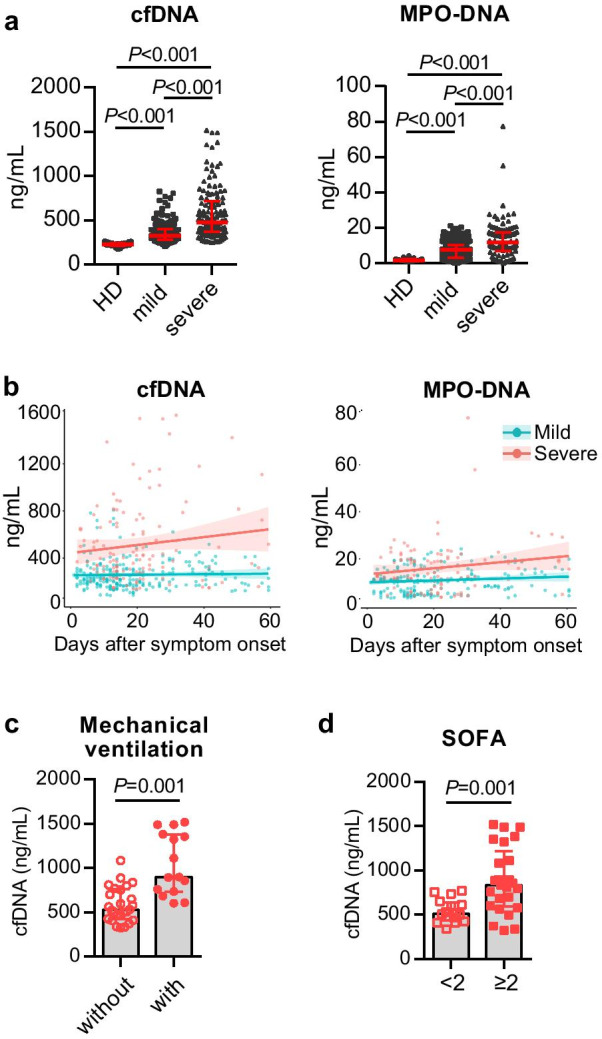
The plasma levels of NETs were associated with severity of COVID-19**. a** Plasma levels of cfDNA and MPO-DNA. HD, *n* = 25; mild, *n* = 94; severe, *n* = 41. **b** Linear regression to straight curves of longitudinal cfDNA and MPO-DNA concentrations in plasma of COVID-19 patients within 60 days after symptom onset. Plasma samples were collected weekly in each patient after admission. Two hundred and twenty plasma samples from mild patients and 119 plasma samples were calculated. Confidence interval at 95% was indicated as a colored shadow. **c** The cfDNA levels in the patients with/without requirement of mechanical ventilation (without, *n* = 12; with, *n* = 15), and the patients with different SOFA scores (< 2, *n* = 22; ≥ 2, *n* = 19) who were admitted to ICU. Data are presented as median (interquartile range). *P* values were obtained by Mann–Whitney U test or Student t test

We collected plasma once a week from hospitalized patients and evaluated the levels of complements and NETs by ELISA. In comparison with HDs, COVID-19 patients had significantly higher levels of NETs in the first week of admission quantified by measuring cell-free DNA (cfDNA) and myeloperoxidase (MPO)-DNA (all *P* < 0.001; Fig. 1a). Linear regression analysis showed that severe patients had higher levels of NETs than mild cases dynamically within 60 days of hospitalization (Fig. 1b). Both cfDNA and MPO-DNA levels in severe cases showed an upward trend with the disease progression (Fig. 1b).

Previous studies have reported that NETs were associated with a variety of pathological changes such as immune status, coagulation disorder, and multiple organ dysfunction [27]. In COVID-19 patients, NET levels were positively correlated with the neutrophil count, fibrin(-ogen) degradation products (FDP), lactate dehydrogenase, and creatine kinase (Table 2). In addition, greater levels of cfDNA were detected in patients with mechanical ventilation (without vs with: 570.4 vs 897 ng/mL, *P* = 0.001; Fig. 1c) or those with sequential organ failure assessment (SOFA) scores ≥ 2 (SOFA < 2 vs SOFA ≥ 2: 554.9 vs 897 ng/mL, *P* = 0.001; Fig. 1d). These results indicated that the NET levels were closely related to the disease severity of COVID-19.

**Table 2:** Correlations between laboratory parameters and the plasma levels of cfDNA, MPO-DNA, C3 and C5 in COVID-19 patients

Parameter	cfDNA		MPO-DNA		C3		C5	
	R	*P*	R	*P*	R	*P*	R	*P*
*Immunological parameters*^*a*^								
WBC (× 10^9^/L)	0.367	** < 0.001**	0.183	0.036	0.190	0.037	0.102	0.265
Neutrophil (× 10^9^/L)	0.439	** < 0.001**	0.305	**0.001**	0.318	**0.001**	0.129	0.159
CRP (U/L)	0.374	** < 0.001**	0.065	0.515	0.194	0.040	0.205	0.029
*Coagulation parameters*^*b*^								
D-dimer (g/L)	0.347	**0.007**	0.2442	0.043	0.240	0.024	0.139	0.196
PT (s)	0.615	** < 0.001**	0.073	0.484	− 0.0043	0.686	0.139	0.190
FDP (μg/mL)	0.474	** < 0.001**	0.316	**0.002**	0.300	**0.005**	0.068	0.526
PTA (%)	− 0.585	** < 0.001**	− 0.0725	0.4851	0.041	0.704	− 0.148	0.164
INR	0.588	** < 0.001**	0.073	0.480	− 0.042	0.695	0.144	-.175
*Tissue damage parameters*^*a*^								
LDH (U/L)	0.379	** < 0.001**	0.306	**0.003**	0.162	0.100	0.143	0.149
DBIL (µmol/L)	0.496	** < 0.001**	0.009	0.367	0.303	**0.004**	− 0.034	0.755
TBIL (µmol/L)	0.360	** < 0.001**	0.054	0.599	0.304	**0.005**	− 0.072	0.507
CK(U/L)	0.309	** < 0.001**	0.308	**0.004**	0.074	0.506	− 0.104	0.349
GFR (g/L)	− 0.389	** < 0.001**	− 0.187	0.165	− 0.333	0.002	− 0.129	0.236

Correlations were calculated by Spearman correlation analysis (r). a, the immunological parameters and tissue damage parameters were analyzed in all enrolled patients (*n* = 135); b, coagulation parameters were measured in 60 patients according to the needs of clinical examination. Mild, *n* = 19; Severe, *n* = 41. Abbreviations: WBC, white blood cells; CRP, C-reactive protein; PT, thrombin time; FDP, fibrin(-ogen) degradation products; PTA, prothrombin activity; INR, international normalized ratio; LDH, lactate dehydrogenase; DBIL, direct bilirubin; TBIL, total bilirubin; CK, creatine kinase; GFR, glomerular filtration rate

*P* < 0.05 was considered statistically significant and is shown in bold

### Increased plasma levels of complement C3 and C5 were positively associated with disease severity in COVID-19 patients

**Fig. 2:**
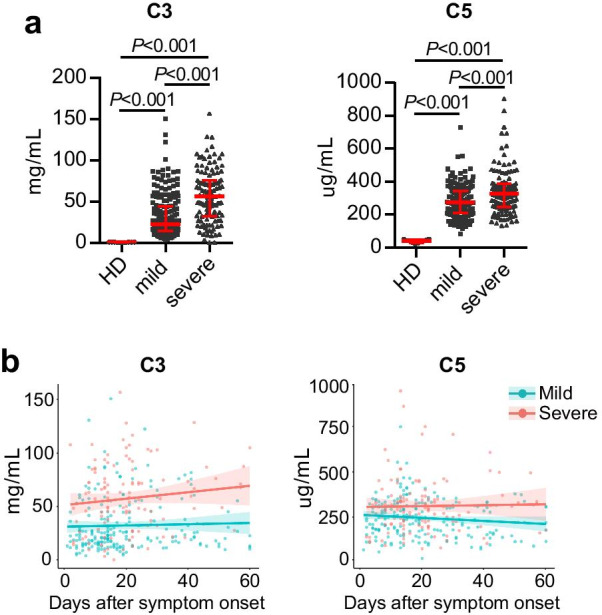
The plasma levels of complement C3 and C5.** a** Plasma levels of C3 and C5. HD, *n* = 25; mild, *n* = 94; severe, *n* = 41. **b** Linear regression to straight curves of longitudinal C3 and C5 concentrations in plasma of COVID-19 patients within 60 days after symptom onset. Plasma samples were collected weekly in each patient after admission. Two hundred and twenty plasma samples from mild patients and 119 plasma samples were calculated. Confidence interval at 95% was indicated as a colored shadow. *P* values were obtained by Mann–Whitney U test or Student t test

We measured the levels of complement C3 and C5 in the plasma of patients and HDs. Within the first week of admission, both of C3 and C5 were dramatically increased in mild patients with COVID-19 compared with HDs (Median with interquartile range, C3: 0.75 [0.65, 0.79] mg/mL vs 22.61 [14.86, 44.22] mg/mL, *P* < 0.001; C5: 41.66 [30.19, 47.7] μg/mL vs 274.8 [209.3, 344.7] μg/mL, *P* < 0.001; Fig. 2a). The levels of C3 and C5 were further raised to 56.23 [31.91, 76.04] mg/mL and 326.1 [245, 388.8] μg/mL in the severe COVID-19 patients (Fig. 2a). Consistent with the longitudinal changes of NETs, linear regression analysis showed that severe patients had higher levels of C3 and C5 than mild cases as the disease progresses (Fig. 2b). There was an upward trend of C3 in patients with disease progression (Fig. 2b). Furthermore, C3 levels were positively correlated with the neutrophil count, FDP, direct bilirubin, and total bilirubin in COVID-19 patients (Table 2).

### The anaphylatoxin-induced NET formation was restrained by recombinant CPB

**Fig. 3:**
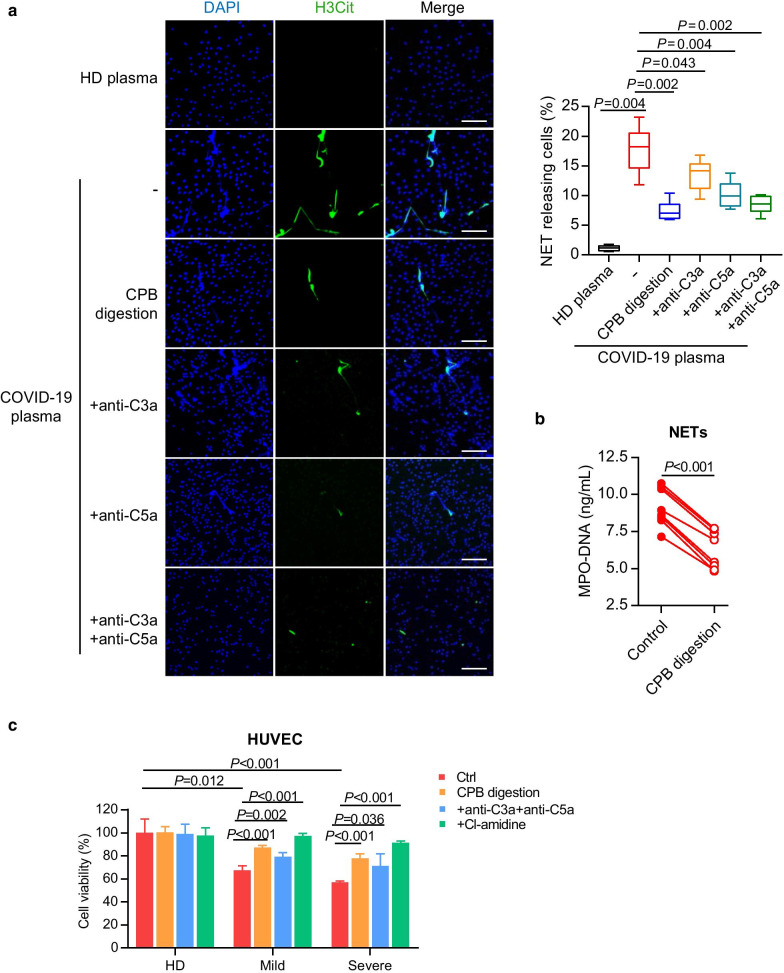
Anaphylatoxin-induced NET release was inhibited by recombinant CPB.** a** Immunofluorescent staining for NET formation. Neutrophils from HDs were cultured with plasma from HDs or severe COVID-19 patients with different treatment. Plasma was digested with 100 μg/ml recombinant CPB for 30 min at 37 °C prior to neutrophil stimulation. One hour prior to neutrophil stimulation, 100 μg/ml anti-human C3a and/or 10 μg/ml anti-human C5a antibodies were added into the culture system for neutralizing C3a or C5a in the plasma. The extracellular histone of NETs was stained with anti-histone H3 citrulline (H3Cit) antibody (green) and DNA was stained with DAPI (blue). Figure shows a representative image from 1 of 3 patients. Scale bar = 100 μm. The percentage of NETs was calculated as an average of 5–10 fields normalized to the total number of neutrophils, and results are expressed as mean ± SEM. **b** NET release as indicated by MPO-DNA concentration in the media. **c** Cell viability of HUVEC cultured with NET-conditioned medium was measured by CCK-8 assay. Cells cultured with HD plasma-conditioned medium were taken as control and normalized as 100%. The peptidylarginine deiminase inhibitor Cl-amidine was added at 200 μM for blocking NET formation. Data are presented as mean ± SEM. Data are presented as median (interquartile range). *P* values were obtained by Mann–Whitney U test or Student t test

To determine the regulation of complement anaphylatoxin C3a and C5a on NET formation, we isolated peripheral neutrophils from HCs (Additional file 1: Figure S1, and Additional file 2: Figure S2a), then cultured these cells in the presence of the plasma from severe patients with COVID-19 or the plasma from HDs. The COVID-19 plasma was capable of activating neutrophils to form NETs (Fig. 3a and Additional file 2: Figure S2b) and to release MPO-DNA in the media (Fig. 3b), which were significantly restrained by anti-C3a and anti-C5a neutralizing antibodies. Moreover, we added recombinant CPB, a stable homolog of enzyme CPB2 which is capable of inactivating anaphylatoxin C3a and C5a by specifically degrading their arginine residues [29]. As shown in Fig. 3a, CPB significantly reduced NET release induced by the plasma from COVID-19 patients. Consistently, we treated neutrophils with recombinant C3a and C5a for 3 h and found that both C3a and C5a could significantly promoted NET release (Additional file 3: Figure S3). These data indicated that the over-production of NETs in the plasma of COVID-19 patients at least in part was induced by anaphylatoxin C3a and C5a, and the induction could be blocked by recombinant CPB.

To further investigate the pathological roles of the anaphylatoxin-NET axis, we stimulated neutrophils with COVID-19 plasma to prepare a NET-conditioned medium and then added the conditioned medium to the culture system of vascular endothelial HUVEC cells. Compare with the plasma from HDs, the plasma from mild and severe COVID-19 patients reduced cell viability of HUVEC cells (Fig. 3c). However, the cytotoxicity of COVID-19 plasma was significantly relieved after in vitro digestion of plasma by CPB for 30 min (Fig. 3c). Similarly, the supplement of anti-C3a antibody plus anti-C5a antibody, or the NET inhibitor Cl-amidine to the cultured neutrophils to block NET production, could also reduce the cytotoxicity to HUVEC cells (Fig. 3c). These results suggested that recombinant CPB could protect vascular endothelial cells from damage by reducing C3a- and C5a-induced NET production in COVID-19 patients.

## Discussion

As one of the major causes of mortality in severe COVID-19, thrombosis has drawn much attention [30]; however, the formation mechanisms remain to be clarified. Recently, NETs were found in to be a key, which provides new clues to the pathogenesis [20, 31–33]. In this study, we investigated the longitudinal dynamics of complement C3, C5 and NETs in the plasma of mild and severe patients with COVID-19. Considering that the average hospitalization periods of mild and severe patients were 25.5 (17.3, 35.7) and 36 (22, 43), respectively (Table 1), an observation of up to 60 days in the present study would cover the dynamics of NETs and complements during the disease progression from onset to convalescence. First, we found that the elevated levels of NETs and complement C3 were closely related to immune status, coagulation disorders, and multiple organ dysfunction. Second, the NET formation was at least partially regulated by complement anaphylatoxin C3a and C5a. Third, we made a novel finding that recombinant CPB could effectively improve the detrimental effect of NETs on vascular endothelial cells by degrading C3a and C5a.

The roles and mechanisms of complement C5a in neutrophil migration and activation have been well described in several inflammatory disorders such as sepsis, inflammatory arthritis and gout [34–38]. In the global pandemic COVID-19, over-activation of complement had also attracted the attention of scientists. Consistent with our study, other reports also revealed that activation of complement C3 and C5 was involved in the pathogenesis of COVID-19 [39, 40]. The findings from our study and these reports collectively suggested that complement activation may contribute to the development of tissue injury and organ dysfunction in patients with COVID-19. Given this, complement-blocking drugs may be a beneficial addition to the therapeutic armamentarium against COVID-19. Thus, several clinical trials were launched just recently in order to prevent ARDS and mortality of COVID-19 by C5a inhibitor (Zilucoplan, NCT04382755) or anti-C5a antibody (Eculizumab, NCT04288713). Considering that in addition to C5a, a significant elevation of C3a was also observed in the patients with COVID-19, we highly recommend recombinant CPB as a potential choice for simultaneously degrading both C3a and C5a[12]. It is worthy of expectation for preclinical studies on recombinant CPB to suppress the unrestrained inflammation and reduce the clinical severity of COVID-19. Notably, the endogenous CPB2 was also known as thrombin-activatable fibrinolysis inhibitor (TAFI) to inhibit fibrinolysis and thereby reduce the binding of plasminogen to the fibrin clot [24]. An excessive supplement of recombinant CPB may upset the balance between coagulation and fibrinolysis. Therefore, an appropriate dosage should be carefully considered in further studies.

We noticed that the NET production induced by plasma from severe COVID-19 patients could not be completely inhibited by either neutralizing antibodies or recombinant CPB (Fig. 3a), which implied that in addition to anaphylatoxins, there were other inducers of NETs in the plasma of severe patients. Many studies have reported an increase in IL-6, IL-1β, and CXCL-8 in severe patients with COVID-19 [41–43], which are also important factors that induce granulocyte activation and NET release. These pro-inflammatory cytokines may also contribute to the over-production of NETs in severe COVID-19 patients. Anaphylatoxins are leading mediators for rapidly inducing the synthesis of pro-inflammatory cytokines [44, 45]. Thus, complement activation may be a pivotal link in amplifying inflammatory response in early infection. Consistently, we found that compared with HDs, complement C3 and C5 increased remarkably in patients with mild symptoms. Although the complement component anaphylatoxins may contribute to increased disease severity following SARS-CoV-2 infection, complement activation is necessary for the development of a protective humoral response. In this respect, early intervention in anaphylatoxins without affecting complement cascade activation in COVID-19 patients might help prevent thrombosis and disease progression.

In addition, C3 and C5 concentrations might be influenced by a variety of factors including confounding co-morbidities. By analyzing the relationship between complement levels and complications, we found that the patients with high levels of C-reactive protein (> 5) had higher concentrations of C3 and C5 in both mild and severe patients (Additional file 4: Figure S4). Meanwhile, the immune status (peripheral neutrophil counts) was correlated with C3 concentrations in COVID-19 patients (Table 2). These data indicated that complement activation was tightly associated with inflammation and immune status in COVID-19.

Our results demonstrated that the increased complement component plays an important role in promoting the formation of NETs in patients with COVID-19. It is different from our previous findings in the infection of severe fever with thrombocytopenia syndrome virus (SFTSV). The patients with SFTS had significantly higher levels of NETs but comparable levels of C3 and C5 to the healthy controls [27]. The difference in complement activation between COVID-19 and SFTS might be related to differences in clinical manifestations. Pulmonary thrombosis appears to be frequent in COVID-19 pneumonia, while the patients with SFTS have a marked propensity for bleeding with a rare thrombus. Thus, the mechanism and function of NETs may be different in these two viral infections associated with coagulation abnormalities.

We acknowledged that our study has several limitations. First, the CPB2 levels in the plasma of COVID-19 patients were not available because it is unstable in physiological condition with a half-life of 10 min at 37℃. Second, for the same reason, we used recombinant pancreatic enzyme CPB instead in in vitro study, which is a stable homolog of CPB2. Third, compared with MPO-DNA, a specific marker of NETs, cfDNA could also be released from other leukocytes and damaged endothelial cells following cellular death. We were not able to accurately determine the cellular origins of peripheral cfDNA in the present study. As there were higher degrees of correlation of cfDNA with clinical parameters than MPO-DNA (Table 2), it may be directly related to leukopenia and tissue damage in patients with COVID-19.

## Conclusions

In conclusion, our study offers new insights into the immunological pathogenesis of COVID-19. Based on these findings, degrading the over-generated C3a and C5a by recombinant CPB to restrain the downstream NET production might be a promising approach to prevent thrombosis and reduce the clinical severity of COVID-19.

## Supplementary information

**Additional file 1: Supplementary figure 1.** Neutrophil morphology and purity.

**Additional file 2: Supplementary figure 2.** Negative and Positive controls of the immunofluorescent staining for NET formation.

**Additional file 3: Supplementary figure 3.** C3a and C5a induced NET formation.

**Additional file 4.** C3 and C5 concentrations in patients with different levels of C-reactive protein (CRP).

**Additional file 5.** Supplementary figure legends.

## Data Availability

All data generated or analyzed during this study are included in this published article.
