# Supplementary figures and images for: Carboxypeptidase B blocks ex vivo activation of the anaphylatoxin-neutrophil extracellular trap axis in neutrophils from COVID-19 patients
Source: Crit Care. 2021 Feb 8;25:51. doi: 10.1186/s13054-021-03482-z (PMC7868871; doi:10.1186/s13054-021-03482-z)

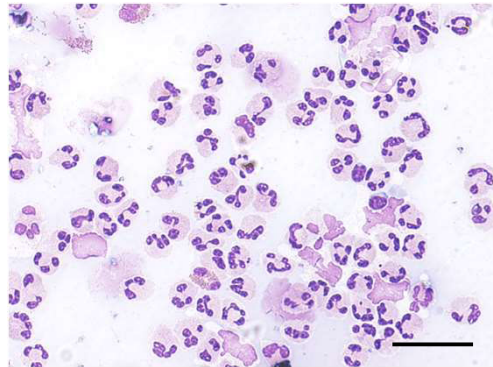

Additional file 1

Supplement: Supplementary file 1 — Additional file 1: Supplementary figure 1. Neutrophil morphology and purity. [file 13054_2021_3482_MOESM1_ESM.pdf]

**A**

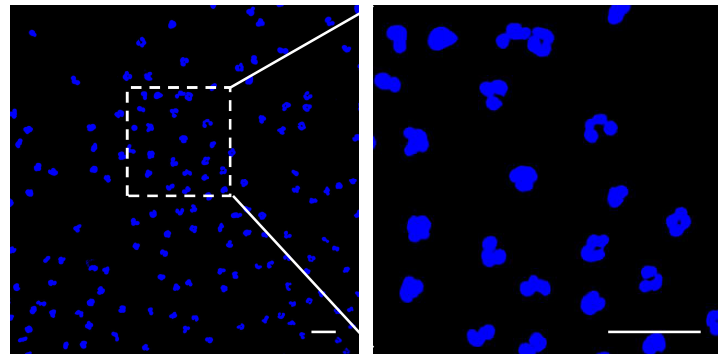

**B**

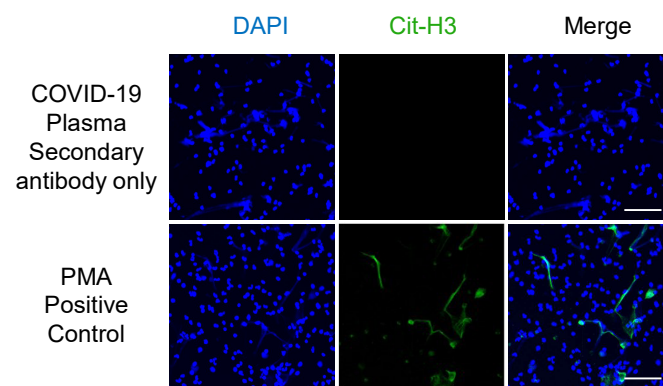

Additional file 2

Supplement: Supplementary file 2 — Additional file 2: Supplementary figure 2. Negative and Positive controls of the immunofluorescent staining for NET formation. [file 13054_2021_3482_MOESM2_ESM.pdf]

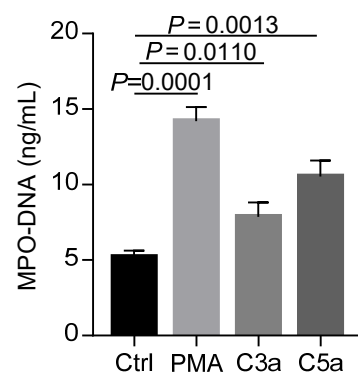

Additional file 3

Supplement: Supplementary file 3 — Additional file 3: Supplementary figure 3. C3a and C5a induced NET formation. [file 13054_2021_3482_MOESM3_ESM.pdf]

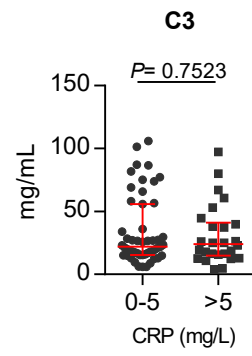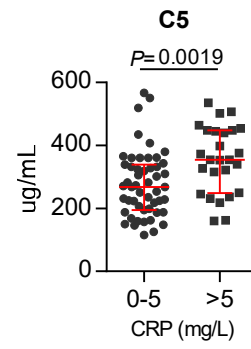

Additional file 4

Supplement: Supplementary file 4 — Additional file 4. C3 and C5 concentrations in patients with different levels of C-reactive protein (CRP). [file 13054_2021_3482_MOESM4_ESM.pdf]
