# Supplementary material for: Carboxypeptidase B blocks ex vivo activation of the anaphylatoxin-neutrophil extracellular trap axis in neutrophils from COVID-19 patients
Source: Crit Care. 2021 Feb 8;25:51. doi: 10.1186/s13054-021-03482-z (PMC7868871; doi:10.1186/s13054-021-03482-z)
Supplement: Supplementary file 5 — Additional file 5. Supplementary figure legends. [file 13054_2021_3482_MOESM5_ESM.doc]

**Additional file figure legends**

**Figure S1. Neutrophil morphology and purity.** Wright Giemsa staining of peripheral neutrophils separated from healthy donors. Neutrophil preparations were at least 95% pure as confirmed by nuclear morphology. Figure shows a representative image from one of three cases. Scale bar = 20 µm.

**Figure S2. Negative and Positive controls of the immunofluorescent staining for NET formation.** (A) Neutrophils from healthy donors were cultured with plasma from severe COVID-19 patients for 3 hours. The negative control cells were exclusively incubated with secondary antibody and DNA was stained with DAPI (upper panel). Neutrophils from healthy donors were cultured with PMA (Sigma, 250 nM) for 3 hours as a positive control (lower panel). The citrullinated histone H3 was detected with specific antibody and shown as green, DNA was stained with DAPI and shown as blue. The dual-labelled immunofluorescent staining is shown as a merged figure. Figure shows a representative image from one of three cases. Scale bar = 100 µm. (B) Magnified pictures of DAPI and Cit-H3 stained neutrophils from HDs cultured with plasma from HDs for 3 hours (Figure 3A, Upper row). Scale bar = 20 µm.

**Figure S3.** **C3a and C5a induced NET formation.** Neutrophils from HDs were cultured with PMA (Sigma, 250 nM), C3a (R&D systems, 200 nM) and C5a (PeproTech, 5nM) for 3 hours, respectively. Cell culture media were collected and detected for the concentrations of MPO-DNA complex. Data are presented as mean ± standard deviation. *P* values were obtained by unpaired *t*-test and Mann-Whitney test.

**Figure S4. C3 and C5 concentrations in patients with different levels of C-reactive protein (CRP).** Data were collected in patients within the first week of admission. CRP 0-5, n = 64; CRP >5, n =71. Data are presented as median (interquartile range). P values were obtained by Mann-Whitney U test.
